# Emerging role of microtubule-associated proteins on cancer metastasis

**DOI:** 10.3389/fphar.2022.935493

**Published:** 2022-09-14

**Authors:** Onsurang Wattanathamsan, Varisa Pongrakhananon

**Affiliations:** ^1^ Preclinical Toxicity and Efficacy Assessment of Medicines and Chemicals Research Unit, Chulalongkorn University, Bangkok, Thailand; ^2^ Department of Pharmacology and Physiology, Faculty of Pharmaceutical Sciences, Chulalongkorn University, Bangkok, Thailand

**Keywords:** cancer, epithelial to mesenchymal transition, metastasis, microtubules, microtubule-associated protein, migration and invasion

## Abstract

The major cause of death in cancer patients is strongly associated with metastasis. While much remains to be understood, microtubule-associated proteins (MAPs) have shed light on metastatic progression’s molecular mechanisms. In this review article, we focus on the role of MAPs in cancer aggressiveness, particularly cancer metastasis activity. Increasing evidence has shown that a growing number of MAP member proteins might be fundamental regulators involved in altering microtubule dynamics, contributing to cancer migration, invasion, and epithelial-to-mesenchymal transition. MAP types have been established according to their microtubule-binding site and function in microtubule-dependent activities. We highlight that altered MAP expression was commonly found in many cancer types and related to cancer progression based on available evidence. Furthermore, we discuss and integrate the relevance of MAPs and related molecular signaling pathways in cancer metastasis. Our review provides a comprehensive understanding of MAP function on microtubules. It elucidates how MAPs regulate cancer progression, preferentially in metastasis, providing substantial scientific information on MAPs as potential therapeutic targets and prognostic markers for cancer management.

## Introduction

Microtubules are important intracellular cytoskeletons that have highly dynamic structural filaments. They are hollow cylindrical structures assembled from 13 aligned protofilaments of tubulin heterodimers (Chaaban and Brouhard, 2017). The biological functions of microtubules are essential in many indispensable cellular activities, including cell division, growth, and movement (Hookway et al., 2015; Prosser and Pelletier, 2017; Singh et al., 2018). These hollow cylinder tubes are structurally rigid and, upon microtubule assembly, provide intracellular structures (Schaedel et al., 2015). The dynamic instability of the microtubule persistently alternates between polymerization and depolymerization phases (Matov et al., 2010). Mechanistically, adding GTP-tubulin dimers to the microtubule plus-end (the forming of a stabilizing cap) is the growing state. Loss of the stabilizing GTP cap, the GDP-tubulin dimers are presented, leading to the switching from a growing state to rapid shrinking (Bowne-Anderson et al., 2015). This dynamic switching activity of microtubules is associated with microtubule-associated proteins (MAPs), which bind to various microtubule sites (Akhmanova and Steinmetz, 2015). Several MAPs are coordinated with molecular trafficking along the microtubules in cells (Seiberlich et al., 2015; Bodakuntla et al., 2019).

The dynamic instability of microtubules plays a role in several cancer activities, such as cell proliferation and cell invasion, which are considered potential cancer therapy targets (Pagano et al., 2012; Li and Wang, 2020). MAPs stabilize microtubules and promote their assembly (Barbier et al., 2010; Breuzard et al., 2013). Interestingly, the dynamic properties of microtubules are indispensable for cell motility (Etienne-Manneville, 2013). Consequentially, MAPs are fundamental factors for cell motility through their regulation of microtubule dynamics (Bodakuntla et al., 2019). Alterations in MAP gene transcription and protein expression were found in many cancer types and were mostly related to cancer metastasis (Wang W. et al., 2020; Zhang et al., 2020). Nowadays, targeting MAPs has been regarded as a strategy in cancer drug research and development. In this review, we summarize currently available knowledge on MAPs, and update and discuss our information on the relationship between several types of MAPs and cancers, owing to their contribution to cancer metastasis.

## Microtubule-associated protein classification

**FIGURE 1:**
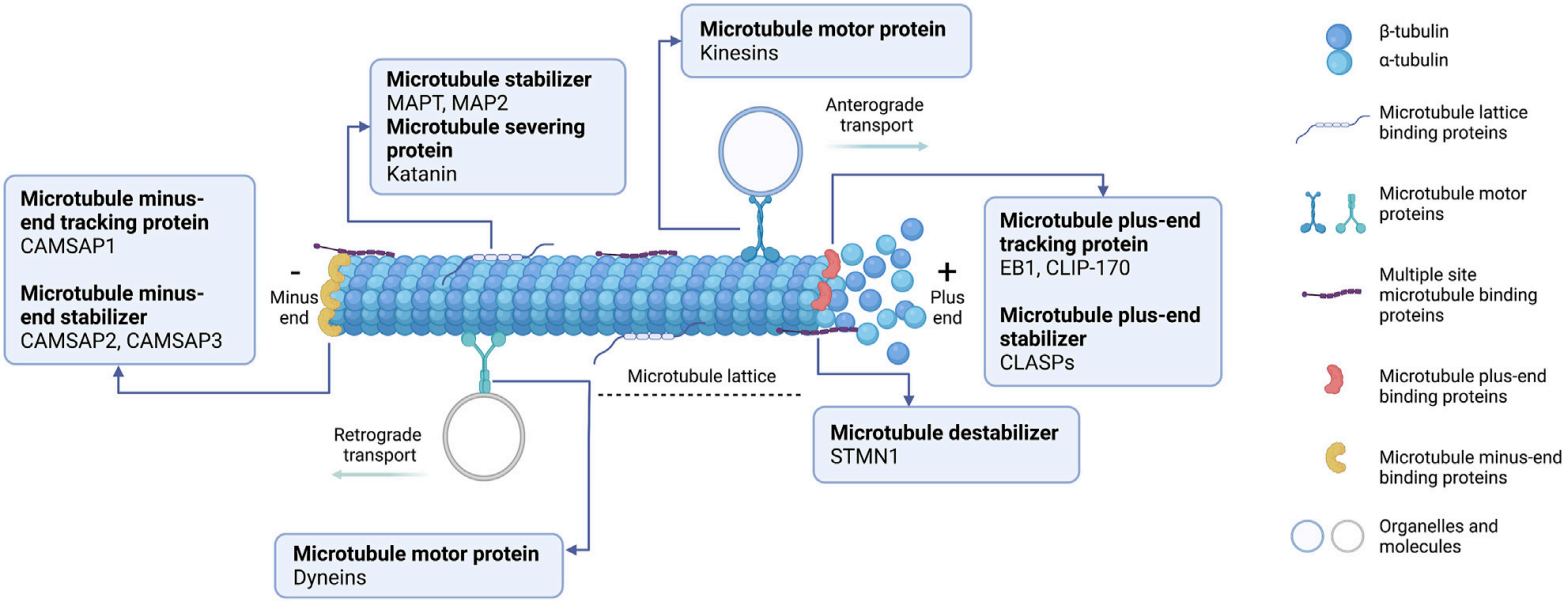
Schematic representation of microtubule-associated proteins (MAPs) binding to microtubules at different sites. (1) Microtubule lattice-binding proteins located at the middle region along the microtubule length, including MAPT and MAP2. (2) Two types of microtubule motor proteins exist: kinesins and dyneins. Kinesins carry the cellular organelle or molecules trafficking towards the microtubule plus-end (anterograde transport). Dyneins transport the cellular molecules to the microtubule minus-end (retrograde transport). (3) Multiple site microtubule-binding proteins, such as stathmin (STMN1), bind to several sites on microtubules. (4) Microtubule plus-end binding proteins (+TIPs) accumulate specifically at the growing ends of microtubules. +TIPs can affect microtubule positioning and regulate microtubules dynamically. (5) Microtubule minus-end binding proteins are preferentially anchored at the minus-end where microtubules are stabilized. This diagram was created with “Biorender.com”.

Microtubules have a critical role in cellular processes, including cell division, cell motility, and cellular trafficking of molecules and organelles (Prosser and Pelletier, 2017; Singh et al., 2018). An assembly of microtubules forms distinct arrays through dynamics and a defined architecture to accomplish the diverse roles of microtubules. Microtubule assembly requires distinctive microtubule-binding proteins, known as MAPs. Based on their localization on the microtubules, MAPs can be categorized into five groups (Figure 1): 1) microtubule lattice binding proteins are located along the microtubule length; 2) microtubule motor proteins are required for cellular transportation along the microtubule; 3) multiple site-binding proteins; 4) microtubule plus-end binding proteins, which are particularly localized to the fast-growing + TIP network of a microtubule; 5) minus-end binding proteins are associated with the slow-growing cap of microtubules (Akhmanova and Hoogenraad, 2015). Moreover, MAPs can be classified functionally into microtubule stabilizers, destabilizers, capping, and bundlers/cross-linker proteins (Goodson and Jonasson, 2018). Broadly speaking, MAPs have multiple and complex activities, making it challenging to conclude their functions because they depend on experimental aspects or assay conditions. Further details about MAPs according to their microtubule localization and activities will be specifically discussed below.

### Microtubule lattice binding proteins

The microtubule lattice is typically composed of 13 protofilaments in its main body (Cross, 2019). In the middle region of the microtubule, the lattice in the GDP stage can be stabilized through classical MAPs’ activity, including microtubule-associated protein tau (MAPT). It is mainly abundant in neuronal axons, regulates microtubule assembly by inhibiting tubulin dissociation, and protects microtubules against depolymerization (Qiang et al., 2006). Similarly, microtubule-associated protein 2 (MAP2) is highly expressed in neuronal dendrites and stabilizes microtubule growth by crosslinking microtubules with intermediate filaments resulting in catastrophe rescue and microtubule stiffness activation (Teng et al., 2001). In contrast, the GDP microtubule lattice can be destabilized by the microtubule-severing protein katanin, a heterodimer protein composed of katanin P60 and katanin P80 subunits, which utilize ATP for severing existing microtubules (Ghosh et al., 2012). Mechanistically, katanin exerts its microtubule-severing function by deploying ATP hydrolysis to extract tubulin dimers at the lattice and disassemble the polymer. Consequently, new microtubule ends are created through a severe lack of GTP caps, and microtubules are rapidly depolymerized (Zehr et al., 2017). Furthermore, angiotensin II (AT2) receptor-interacting protein 3 (ATIP3) is one of the structural MAP localized along the microtubule lattice and is a potent microtubule stabilizer by associating with EB1, thereby attenuating microtubule dynamics (Nehlig et al., 2021). Comprehensively, ATIP3 interacts with EB1 in the cytosol, and the ATIP3-EB1 complex regulates the microtubule growth-shrinkage rate by preventing the accumulation of EB1 at the plus end (Velot et al., 2015).

### Microtubule motor proteins

Intracellularly, molecular transport can be carried out along microtubule tracks using motor proteins, especially kinesins and dyneins (Lu and Gelfand, 2017). Kinesin motor proteins “walk” in a specific anterograde direction and intracellular transport cargoes toward microtubule plus-ends (Kevenaar et al., 2016). Kinesins are composed of different subtypes of their family proteins involved in individual cellular activities. For example, the classical kinesins, including kinesins 1 (KIF5B), 2 (KIF3A/B, 17), 3 (KIF13B), 5 (KIF11), 6 (KIF6, 9), and 8 (KIF19A, 18B), with an N-terminal motor domain and KIF14, with a middle motor domain, deploy ATP hydrolysis to generate kinesin motor movement in mechanical force toward growing microtubule plus-ends (Gigant et al., 2013; Arora et al., 2014). Furthermore, kinesin-14 or KIFC1 has a similar role as classical kinesins but has a different C-terminal motor domain, which it transports several cargos toward the microtubule minus-ends (She et al., 2017). Finally, the middle motor domain kinesin-13 or KIF2A has no motile activity, unlike other kinesin types, and has a depolymerizing microtubule activity initiated by the motor domain (Trofimova et al., 2018).

Another motor protein type, dyneins, can move toward the microtubule minus-end and transport intracellular cargoes from the cellular periphery to the center in a retrograde direction transport (Roberts et al., 2013). This motor protein guides the Golgi complex or other organelles and positions the mitotic spindle in cell division (Roberts et al., 2013). Cytoplasmic dynein is a large multi-subunit protein forming a complex called dynactin associated with the dynein motor to enhance its processivity for cargo transportation (Chowdhury et al., 2015). Because of these different functions, kinesin and dynein play important roles in distinct microtubule-dependent activities, organelle transportation, intracellular vesicle trafficking, and mitotic spindle organization (Cross and McAinsh, 2014; Kevenaar et al., 2016; Tan et al., 2018).

### Multiple site microtubule-binding proteins

The regulation of microtubule dynamic instability is influential for cellular processes. One of the most remarkable microtubule destabilizers is stathmin (STMN1), or oncoprotein 18 (Op18) (Zeitz and Kierfeld, 2014). STMN1 efficiently promotes catastrophe through microtubule-binding activities, in which the STMN1-microtubule interaction occurs via various molecular mechanisms (Gupta et al., 2013). STMN1 is a sequestering protein that reduces microtubule polymer length by indirectly binding to tubulin subunits in a curved form, promoting depolymerization (Zeitz and Kierfeld, 2014). Moreover, STMN1 also induces microtubule destabilization by specifically interfering with the lateral binding of tubulin subunits (steric inhibition or by inducing curvature) at microtubule tips, operating both on plus- and minus-ends (Gupta et al., 2013). As a minus-end mechanism, the stathmin N-terminal peptide binds to and caps the dimer of α-tubulin that is exposed and prevents the incorporation of new tubulin dimers, resulting in catastrophe (Gupta et al., 2013). Additionally, the binding of STMN1 to the microtubule lattice can contribute to catastrophe, but is less effective than other mechanisms (Barbier et al., 2010). The STMN1-tubulin direct mechanism and STMN1-tubulin sequestering phenomenon can function together to create STMN1’s cellular activities.

### Microtubule plus-end binding proteins

Microtubule dynamics are spatially mediated by MAPs, which preferentially contain polymerizing microtubule plus-ends and are known as plus-end tracking proteins (+TIPs) (Matov et al., 2010). Several + TIPs regulate microtubule dynamics, act as adaptors connecting microtubule plus-ends to other factors or cellular components, and promote microtubule assembly to membrane transportation (Galjart, 2010). The first identified + TIP protein was cytoplasmic linker protein 170 (CLIP-170). It participates in binding endocytic vesicles to microtubules (Folker et al., 2005). This + TIP protein has CAP-Gly domains that potentially bind to C-terminal EEY motifs, as present in the α-tubulin acidic tail and another + TIP protein, EB1 (Chen L. et al., 2019). Additionally, the N-terminal CAP-Gly domain of CLIP-170, in residues 1–350, tracks a polymerizing microtubule plus-ends in an EB1-dependent manner (Dixit et al., 2009). End binding proteins are another central + TIP protein type, including EB1, EB2, and EB3. They can precisely bind to the plus-end of microtubules and associate with the stabilizing GTP cap of microtubule growth (Nehlig et al., 2017). Likewise, Cytoplasmic Linker-Associated Proteins (CLASPs) are a conserved class of +TIPs proteins that contribute to microtubule stabilization (Lindeboom et al., 2019). A recent study proposed the rescue mechanism of CLASPs. They form a complex with tubulin dimers and bind along the microtubule lattice resulting in reverse microtubule depolymerization (Aher et al., 2020).

### Microtubule minus-end binding proteins

Various MAPs play a crucial role in regulating microtubule dynamics at their ends, including calmodulin-regulated spectrin-associated proteins (CAMSAPs), a recently described microtubule minus-end binding proteins (Hendershott and Vale, 2014). In mammals, CAMSAP family members consist of CAMSAP1, CAMSAP2, and CAMSAP3. They potentially regulate the stability and localization of microtubule minus-ends and thus organize non-centrosomal microtubule networks, sufficient for cell division, migration, and polarity (Akhmanova and Hoogenraad, 2015; Atherton et al., 2017). In mammals, CAMSAP1 is a growing minus-end tracking protein. CAMSAP2 and CAMSAP3 promote microtubule minus-end polymerization, which initiates the stable microtubule stretches by serving as a seed for non-centrosomal microtubule outgrowth (Akhmanova and Hoogenraad, 2015). These two CAMSAPs form stretches of deposited microtubule lattice, which is highly stable, and prevent microtubule depolymerization from both ends (Atherton et al., 2017). CAMSAP2 and CAMSAP3 are mostly reported concerning microtubule dynamics and organization, providing fundamental anti-cancer drug research and development strategies.

## Microtubule-associated proteins and their role in cancer metastasis

**FIGURE 2:**
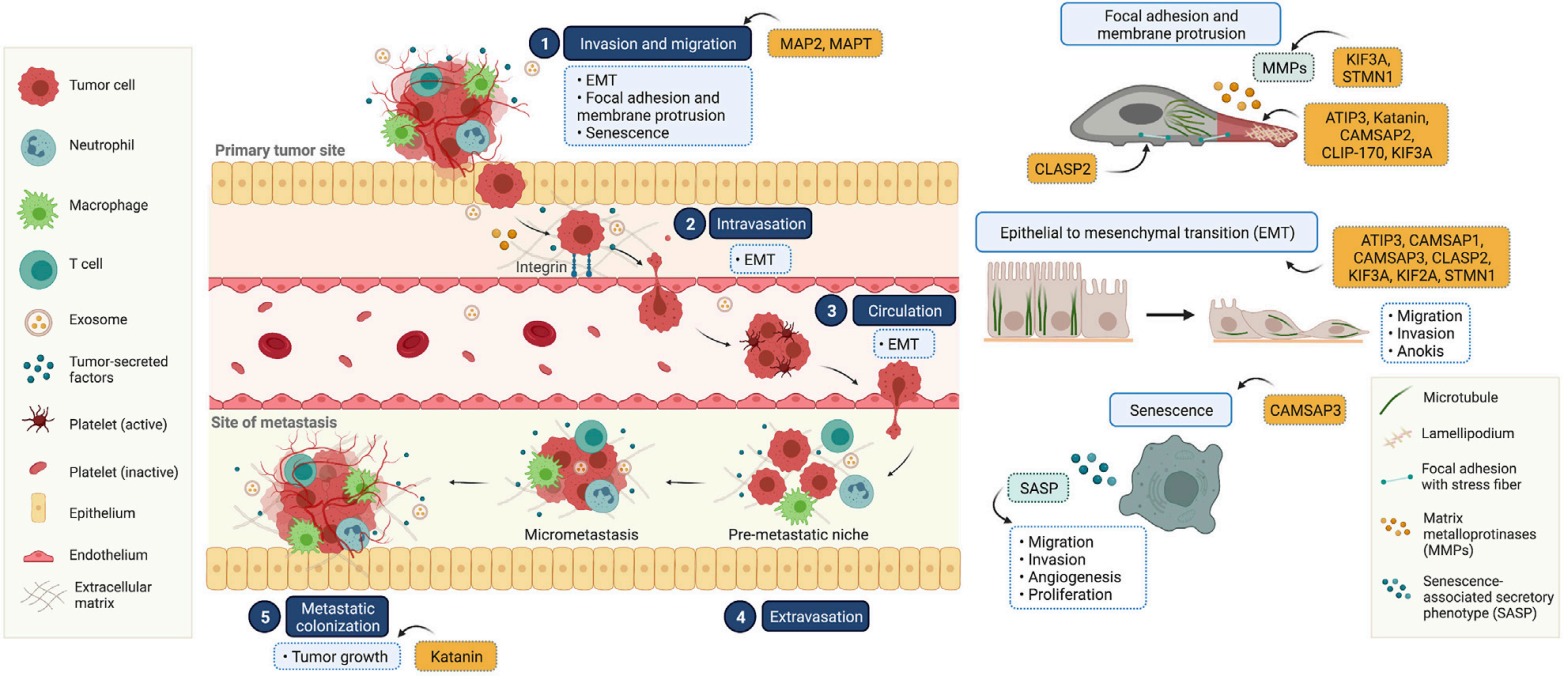
Illustrated overview of the tumor metastatic process and the participation of microtubule-associated proteins (MAPs) in this process. (1) Invasion and migration: cancer cells invade and migrate through the basement membrane and tumor stroma, respectively. This process is involved in epithelial to mesenchymal transition (EMT), focal adhesion and membrane protrusion, and senescence. (2) Intravasation: tumor cells collide with the endothelial membrane and attach firmly via integrin (cell-to-cell adhesion molecules). Subsequently, cancer cells intravasate into the surrounding vasculature or lymphatic system. (3) Circulation: circulating tumor cells survive in the circulating blood and have the ability to evade the immune clearance system. (4) Extravasation: once cancer cells reach their secondary targets, they enter through the endothelial barrier and form a premetastatic niche for tumor cell growth and survival. (5) Colonization: the premetastatic niches recruit several tumor-secreted factors, and the extracellular matrix is remodulated at these sites in a phenomenon known as micrometastasis. In the final step, micrometastasis achieves colonization at the distant organ and secondary tumors are established. This diagram was created with “Biorender.com”.

Cancer metastasis is characterized by multi-step processes in which cancer cells disseminate from their tissue of origin, leading to secondary tumor formation in other organs (Fares et al., 2020) (Figure 2). After primary tumor tissue is formed, an imbalance of nutrients supporting tumor growth mediates vascularization in terms of angiogenesis, facilitating cancer cell detachment, migration, and invasion into nearby blood vessels. In these new environments with growth factor enrichment, cancer cells extravasate and continue growing, completing secondary tumor establishment. In the metastasis process, the reorganization of microtubules through their dynamic properties occurs to support morphological changes associated with cell movement (Fife et al., 2014). Besides the direct functions of microtubules, they also serve as tracking trails for the translocation of metastasis-related signaling molecules to the leading and/or rear edges of cells (Kriebel et al., 2008; Garcin and Straube, 2019). Multiple signaling mechanisms govern these complex steps, which are potential therapeutic targets. Since several MAPs reportedly show differential expression in cancer cells compared to that in normal cells, they likely contribute to cell migration and invasion. Our recent knowledge of MAPs in cancer metastasis has been updated and discussed in (Table 1).

**TABLE 1:** List of microtubule-associated proteins and recent evidence in cancers.

Microtubule-associated proteins	Class	Cancer types	Expression/Mutation in clinical samples	Metastasis	References
Angiotensin II type 2 receptor-interacting protein 3a (ATIP3)	Microtubule lattice binding protein	Breast cancer	Downregulation	Correlates with lymph node metastasis	Rodrigues-Ferreira et al. (2020)
		Breast cancer	-	*In vitro* knockdown promotes cell migration and sensitizes to taxol	Molina et al. (2013)
		Salivary adenoid cystic carcinoma, Tongue squamous cell carcinoma	-	*In vitro* knockdown promotes EMT	Zhao et al., 2015; Zhao et al., 2016
Calmodulin-regulated spectrin-associated proteins 1 (CAMSAP1)	Microtubule minus-end binding protein	Lung cancer	Mutation	Inhibits EMT	Yi et al. (2021)
Calmodulin-regulated spectrin-associated proteins 2 (CAMSAP2)	Microtubule minus-end binding protein	Hepatocellular carcinoma	Upregulation	Promotes cell migration and invasion	Li et al. (2020)
Calmodulin-regulated spectrin-associated proteins 3 (CAMSAP3)	Microtubule minus-end binding protein	Lung cancer	*-*	*In vitro* knockout promotes EMT	Pongrakhananon et al. (2018a)
		Lung cancer		*In vitro* knockout promotes senescence	Wattanathamsan et al. (2021)
Cytoplasmic linker-associated protein 2 (CLASP2)	Microtubule plus-end binding protein	Bladder cancer	Upregulation	Promotes EMT	Chen Y. et al., 2019; Zhu et al., 2017
Cytoplasmic linker protein 170 (CLIP-170)	Microtubule plus-end binding protein	Cervical cancer	-	Promotes cell migration	Zaoui et al. (2019)
		Pancreatic cancer		Promotes cell migration	Li et al. (2014)
Katanin	Microtubule lattice binding protein	Prostate cancer, breast cancer	Upregulation	-	Ye et al., 2012; Zuo et al., 2018
		Lung cancer	Upregulation	Correlates with lymph node metastasis	Wang W. et al. (2020)
		Breast cancer	-	*In vitro* knockdown inhibits cell migration	Fu et al. (2018)
		Colorectal carcinoma	-	Suppresses Tumor growth	Kirimtay et al. (2020)
Kinesin family member 3A (KIF3A)	Microtubule motor protein	Breast, prostate, and bladder cancers	Upregulation	*-*	Xia et al., 2018; Liu et al., 2014; Zhou et al., 2021
		Breast cancer	Upregulation	Correlates with lymph node metastasis, *and in vitro* knockdown inhibits EMT	Wang L. et al. (2020)
		Prostate cancer	-	*In vitro* knockdown inhibits cell migration and invasion	Liu et al. (2014)
Kinesin family member 14 (KIF14)	Microtubule motor protein	Retinoblastoma, ovarian, breast, and gastric cancers	Upregulation	*-*	Madhavan et al., 2007; Thériault et al., 2012; Gerashchenko et al., 2020
		Gastric cancers	Upregulation	Correlates with metastasis, and *in vitro* knockdown inhibits cell migration and invasion	Yang et al. (2019)
microtubule-associated protein 2 (MAP2)	Microtubule lattice binding protein	Gastric adenocarcinoma	Upregulation	Correlates with lymph node metastasis and cancer invasion, and *in vitro* knockdown inhibits cell migration	Zheng et al. (2014)
		Melanoma	Downregulation	Exogeneous MAP2 expression suppresses cell migration and invasion	Song et al. (2010)
Microtubule-associated protein tau (MAPT)	Microtubule lattice binding protein	Glioblastoma	Downregulation	Correlates with a poor survival rate	Zaman et al., 2019
		Renal cell carcinoma	-	*In vitro* knockdown promotes invasion	Han et al. (2020)
Stathmin (STMN1)	Multiple site microtubule-binding protein	Lung cancer, head and neck squamous cell carcinoma, gastric cancer, and colorectal cancer	Upregulation	Correlates with metastasis	Nie et al., 2015; Wu et al., 2018; Liu et al., 2015; Leiphrakpam et al., 2021
		Lung cancer	-	Promote cell migration and invasion	Lu et al. (2014)
		Lung cancer	-	*In vitro* knockdown inhibits EMT	Xun et al. (2021)
		Gastric cancer	-	*In vitro* knockdown inhibits cell migration and invasion	Shu et al. (2019)

### Migration and invasion

Cancer migration and invasion involve motile activity and extracellular matrix degradation. The microtubule dynamics participate in cell movement, and interference with these behaviors affects cancer migration and invasion. MAPT is abundant in neuronal axons and stabilizes microtubules by regulating microtubule polymerization and dynamic behaviors (Barbier et al., 2019). Its function has been extensively investigated in neuronal development and physiology. In neuronal-associated cancer, MAPT overexpression was tightly correlated with an increased overall survival rate in glioblastoma, being considered a tumor suppressor (Zaman et al., 2019). Consistent with renal cell carcinoma, low MAPT expression is associated with a poor overall survival rate, and MAPT RNA interference promotes cancer invasion (Han et al., 2020). Similarly, MAP2 has been identified as a microtubule stabilizer, locally restricted to neuronal dendrites (Harada et al., 2002). Due to its differential expression among neuronal epithelial tumor subtypes and limited expression in neurons, it is a powerful tumor marker (Blümcke et al., 2004; Yi et al., 2018). Among non-neuronal cancers, MAP2 upregulation, compared to adjacent normal tissue, was identified in gastric adenocarcinoma (Zheng et al., 2014). This remarkable upregulation was strongly associated with cancer invasion and lymph node metastasis. Consistently, MAP2 knockdown was shown to impair cancer cell migration (Yang et al., 2020). Contrastingly, in malignant melanoma, MAP2 was rarely expressed in metastasis tumors compared with its expression in primary melanoma (Song et al., 2010). The introduction of MAP2 into metastatic cancer cells interfered with microtubule assembly, leading to the *in vitro* suppression of cell migration and invasion. This suggests that MAP2 expression shows cancer-type specificity. However, the molecular mechanism behind the regulation of cancer metastasis by MAP2 is largely unknown.

As a microtubule dynamic modulator, ATIP3, a microtubule lattice binding protein, regulates breast cancer cell migration through stabilizing microtubules (Molina et al., 2013). Low expression of ATIP3 was correlated with low survival rate and lymph node metastasis in breast cancer patients (Rodrigues-Ferreira et al., 2020). Mechanically, ATIP3 deficiency enhanced microtubule dynamic at the front and rear of the cells, facilitating cell motility. Furthermore, the alteration of microtubule dynamics due to ATIP3 knockdown increased an intracellular accumulation of taxol along microtubules, thereby exerting sensitivity to taxol. This suggests that ATIP3 is a potential target for cancer therapy.

Katanin, a microtubule-severing enzyme, is a heterodimer of P60 and P80 subunits (Ghosh et al., 2012). It plays a fundamental role in regulating microtubule organization, affecting several cellular activities (Zehr et al., 2017). Recent studies have identified the role of katanin in various cancers. Clinical investigations have demonstrated that katanin expression is abundant in cancers, especially in advanced stages (Ye et al., 2012; Zuo et al., 2018). Lung cancer patients with high katanin expression show a poor survival profile, rendering katanin expression as a biomarker for predicting cancer pathogenesis (Wang L. et al., 2020). Katanin upregulation potentially enhanced breast cancer cell migration; conversely, katanin knockdown attenuated this behavior (Fu et al., 2018). The association between microtubule destabilization and cancer metastasis has not been fully elucidated. The exact mechanisms of metastasis regulation by katanin, besides its microtubule-severing properties, are unknown yet.

The translocation of organelles and molecules serves most cell activities, including cell migration. The trafficking of signaling molecules to membrane protrusions is necessary to generate focal adhesions (FAs) to new attachments, followed by organelle movement and cell body contraction, which are required for proper cell motility (Fourriere et al., 2019; Zinn et al., 2019). The coordination of microtubules and their binding proteins plays a crucial role in these events. CLIP-170, a +TIP MAP, is widely known to regulate cellular trafficking through microtubules (Chen Y. et al., 2019). Likewise, CLIP-170 has been reported to regulate the recycling of Met receptor tyrosine kinase (Met RTK) (Zaoui et al., 2019). An internalization of Met RTK is essential for RTK degradation and recycling to the cell surface (Barrow-McGee and Kermorgant, 2014). Disturbance to these processes affected its stability and contributed to tumorigenesis (Xiang et al., 2017). CLIP-170 is required for the endocytic trafficking of Met RTK on microtubules and relocated to the plasma membrane, where lamellipodia, flat sheet-like membrane protrusions, are formed, thereby enhancing cell motility. This study demonstrated the linkage between microtubules, MAP, and endocytic vesicles, which contributes to cancer cell aggressiveness. Nevertheless, the regulation of cellular transport by CLIP-170 was generally observed in both normal and cancer cells. Its specific functions in cancer and expression profile from clinical specimens are being further clarified. The scientific information is currently restricted to support it as a therapeutic target.

Apart from that, CLASP2, a microtubule plus-end binding protein, participates in several microtubule-associated processes (Lindeboom et al., 2019). Given its close relationship with microtubules, CLASP2 reportedly regulated FA turnover, supporting cell migration (Stehbens et al., 2014). It couples with microtubules for transportation of FA components which are required for FA formation and extracellular matrix degradation. CLASP2 expression was significantly increased in muscle-invasive bladder urothelial cancer tissues, particularly in tissues with lymph node metastasis (Chen L. et al., 2019). Its high expression was strongly associated with a poor prognosis. The introduction of exogenous CLASP2 in bladder cancer cell lines exerted *in vitro* cell migration and invasion (Zhu et al., 2017).

Matrix metalloproteinase (MMP) 2 and 9 play an important role in cancer invasion through their extracellular matrix enzymatic degradation. KIF3A was identified as a diagnostic parameter for breast, prostate, and bladder cancers (Liu et al., 2014; Xia et al., 2018; Zhou et al., 2021). It was overexpressed in breast cancer tissues compared to adjacent samples, being particularly associated with lymph node metastasis (Wang W. et al., 2020). RNA interference to KIF3A decreased MMP2 and MMP9 expression, suggesting that KIF3A was required for breast cancer metastasis (Wang L. et al., 2020). KIF3A knockdown in triple-negative breast cancer cell lines significantly attenuated *in vitro* cancer migration and invasion and *in vivo* metastasis (Wang W. et al., 2020). In prostate cancer, KIF3A silencing enhanced cancer migration and invasion through Wnt/β-catenin signaling (Liu et al., 2014). The upregulation of KIF3A activates this pathway by increasing DVL2 phosphorylation and β-catenin levels, promoting MMP9 and HEF1 expression and inducing cancer invasion and migration (Liu et al., 2014). Apart from direct regulation of signaling, KIF3A was shown to interact with APC and β-catenin for intracellular transport via microtubules to the membrane protrusions to exert cell migration (Jimbo et al., 2002).

Besides, proteomic analyses identified several differentially expressed proteins after STMN1 silencing. STMN1 contributes to cancer metastasis, and STMN1 RNA interference significantly inhibits lung cancer cell migration and invasion (Xun et al., 2021). STMN1 knockdown upregulated MMP1 and MMP9, responsible for cancer invasion (Shu et al., 2019). Clusterin, a glycoprotein encoded by the CLU gene, participates in EIF3I/AKT/MMP13 signaling, which facilitates cancer metastasis (Wang et al., 2015). Clusterin was downregulated in response to STMN1 suppression. Furthermore, phosphorylated AKT and STAT were gradually reduced, suggesting that the oncogenic role of STMN1 in cancer metastasis was mediated by clusterin/AKT/MMP and STAT signaling (Shu et al., 2019). Further, STMN1 negatively correlated to tumor suppressor phosphatase and tensin homolog deleted on chromosome 10 (PTEN). High STMN1 expression was detected in lung cancer with low PTEN expression (Xun et al., 2021). PTEN, classified as a tumor suppressor, is a crucial inhibitor of the phosphatidylinositol three kinase (PI3K)/AKT pathway through its phosphatase activity (Milella et al., 2015). Furthermore, PTEN loss enhanced STMN1 upregulation and ameliorated STMN-mediated cancer migration and invasion, indicating that PTEN is an upstream regulator of STMN1. These findings highlighted the interplay between STMN1 and PI3K/AKT pathways. Since cancer metastasis involves complex processes, in which STMN1 has multiple metastasis-regulating targets, STMN1 is a potential target for cancer therapy.

Interestingly, a family of non-centrosomal microtubule minus-end binding proteins, CAMSAPs, was recently identified to govern cancer metastasis. CAMSAPs are consisting of CAMSAP1, CAMSAP2, and CAMSAP3 (Hendershott and Vale, 2014). In the past decade, CAMSAPs have been found to govern neuronal development and epithelial tissue organization (Tanaka et al., 2012; Toya et al., 2016; Pongrakhananon et al., 2018a). However, their functions in cancer remain elusive. Since cancer metastasis is associated with morphological alterations to enhance cell motility, and CAMSAPs control epithelial tissue morphology, it has been hypothesized that CAMSAPs may play a significant role in cancer metastasis. Gene expression analysis obtained from tumor specimens demonstrated that CAMSAP1 mutations, mainly occurring in its CH and CKK domains, significantly correlated with better prognoses in small cell lung cancer, whereas CAMSAP1 expression was not relevant to overall survival rates (Yi et al., 2021). However, available evidence on CAMSAP1’s role in cancer metastasis remains incomplete. In contrast, according to The Cancer Genome Atlas database, significant CAMSAP2 upregulation correlated with poor overall survival rates in several malignancies, including lung squamous cell carcinoma, colon adenocarcinoma, esophageal carcinoma, and pancreatic adenocarcinoma (Chandrashekar et al., 2017). Clinical investigations reported that CAMSAP2 was abundantly expressed in hepatocellular carcinoma and associated with metastasis (Li et al., 2020). The loss of CAMSAP2, as a microtubule stabilizer, caused a transformation of microtubule patterns to radial centrosomal microtubules, whose post-translational acetylation was remarkably reduced. This phenomenon attenuated cancer cell migration and invasion, consistent with a previous study that tubulin acetylation is essential for cell motility (Boggs et al., 2015). Its underlying mechanism was further revealed, whereby CAMSAP2 suppressed histone deacetylase 6 (HDAC6), an enzyme responsible for deacetylation, through a Rac/JNK pathway. Therefore, CAMSAP2 is considered a metastasis promoter. How CAMSAP family members, which share similar conserved regions, govern cancer differently grants further studies.

### Epithelial to mesenchymal transition

The transformation of epithelial to mesenchymal phenotypes plays an essential role in cancer metastasis. A tight organization of epithelial cells with high intercellular adhesion forces alters mesenchymal-like cells with low cell-cell interaction (Datta et al., 2021). The reorganization of the cytoskeleton, including microtubules, contributes to morphological changes to the spindle-like phenotype, facilitating the metastatic ability of cancer. Several MAPs were reported to govern microtubule behaviors and the transportation of signaling molecules involved in the epithelial to mesenchymal transition (EMT) process. In light of the emerging biology of microtubule motor proteins, KIFs play an essential role in the cellular trafficking of components or molecules along microtubules (Hirokawa et al., 2009). Silencing of KIF3A demonstrated that the epithelial to mesenchymal transition was strongly suppressed by the downregulation of a transcription factor, ZEB1, and vimentin, as opposed to the upregulation of the epithelial marker E-cadherin, thereby inhibiting cancer metastasis (Wang L. et al., 2020). However, the molecular mechanism for regulating EMT marker expression by KIF3A remains undiscovered.

Similarly, KIF14 was reported as a potential predictive factor for poor prognosis. It was highly expressed in several cancers including retinoblastoma, ovarian, breast, and gastric cancers (Madhavan et al., 2007; Thériault et al., 2012; Yang et al., 2019; Gerashchenko et al., 2020). Expression analyses from clinical specimens demonstrated a strong correlation between KIF14 expression levels and clinicopathological characteristics, with high expression levels found in metastasis samples (Yang et al., 2019). KIF14 silencing in gastric cancer cells inhibited cell migration and invasion by suppressing the phosphorylation of protein kinase B (AKT), a key mediator for cancer metastasis (Yang et al., 2019). Consistently, there is a molecular mechanism of KIF2A in carcinogenesis (Chen et al., 2022). However, the underlying mechanism of how KIFs control various signaling pathways requires further clarification through motor or other functions.

Stathmin (STMN1), a microtubule destabilizing protein, has gained more attention in cancer pathogenesis in the past decade. Its aberrant overexpression is extensively correlated with poor clinical outcomes in multiple cancers, being particularly associated with metastasis (Liu et al., 2015; Nie et al., 2015; Wu et al., 2018; Leiphrakpam et al., 2021). Based on the classical function, STMN1 mediated microtubule disruption and led to cellular dysfunction (Lu et al., 2014; Zhao et al., 2019). STMN1 reportedly interfered with microtubule stability and mediate EMT (Lu et al., 2014).

Microtubule instability caused by the knockdown of microtubule stabilizer ATIP3 increased an EMT feature (Zhao et al., 2015). ATIP3 restoration could downregulate vimentin and slug and upregulate epithelial E-cadherin marker. Besides affecting microtubule dynamics, ATIP3 functions as a tumor suppressor protein participating in cancer signaling. ATIP3 silencing enhanced extracellular signal-regulated kinase (ERK) phosphorylation, consequently upregulating its downstream target snail and vimentin that promoted the EMT process (Zhao et al., 2016). However, the exact mechanism of how ATIP3 regulates ERK activity was largely unidentified.

The regulation of EMT marker by a microtubule plus-end binding protein was also reported. CLASP2 overexpression mediated the upregulation of vimentin, a mesenchymal marker (Zhu et al., 2017). In contrast, its knockdown resulted in the upregulation of E-cadherin, an epithelial marker. However, the mechanism whereby CLASP2 promotes EMT remained unknown. CLASP2 may govern the nuclear translocation of EMT regulatory transcription factors, which requires further studies. Additionally, the information on CLASP2 was limited only to bladder cancer, and investigations in other malignancies could provide benefits for clinical applications.

Besides, a non-centrosomal microtubule minus-end binding protein CAMSAP3 plays a distinct suppressive role on cancer metastasis, unlike other CAMSAPs. Our group demonstrated that *CAMSAP3* knockout promoted lung cancer cell migration in an AKT-dependent mechanism (Pongrakhananon et al., 2018b). As a fundamental role, CAMSAP3 maintains microtubule stability, and CAMSAP3 depletion contributes to microtubule hyperacetylation, facilitating cancer migration. The association between microtubule acetylation and oncogenic signaling was further identified. In the absence of CAMSAP3, AKT phosphorylation became more stabilized on microtubules, especially acetylated ones. Since AKT signaling is a key driver for EMT, increases in AKT phosphorylation in response to *CAMSAP3* knockout mediated cell migration. However, the mechanism of CAMSAP3’s effect on tubulin acetylation requires further verification.

### Senescence

Generally, cellular senescence is a process involving potentially irreversible growth arrest (Muñoz-Espín and Serrano, 2014). It has been reported that senescence is a therapeutic target for anti-cancer drugs to suppress tumor growth and enhance immune filtration, suggesting a tumor suppressive activity (Bian et al., 2020; Fitsiou et al., 2022). However, senescence plays a dual role in cancers that also acts as an intermediate stage, and secreted senescence-associated secretory phenotypes (SASPs) that were required for cancer metastasis (Capparelli et al., 2012; Guccini et al., 2021). During senescence, microtubule stability appeared to increase since senescence affects both structural and functional changes (Moujaber et al., 2019). The impact of MAPs on senescence was recently reported. CAMSAP3 played a significant role in cellular senescence (Wattanathamsan et al., 2021). CAMSAP3 loss mediated *in vitro* and *in vivo* senescence-like phenotypes and increased SASP expression by suppressing ERK (Wattanathamsan et al., 2021). Besides the involvement of microtubule stabilization, proteomic analysis revealed that vimentin is essential for CAMSAP3-regulated ERK signaling. These findings have shed light on the molecular mechanism of MAPs in cancer senescence. However, an interplay of MAPs in the regulation of microtubule dynamic during senescence was not fully identified.

### Tumor growth and tissue colonization

To complete metastasis, the tumor colonization was established at a distant site in which several growth activators were enriched, as opposed to growth suppressors being attenuated. A tumor suppressor p53 is a key DNA damage sensor preventing uncontrolled cell proliferation. The association of p53 and MAPs in the regulation of cancer growth was reported. p53 binds to the *KATNA1* promoter and positively regulates katanin transcription in human colorectal carcinoma cells. Furthermore, this activity is more pronounced under hypoxic conditions (Kirimtay et al., 2020). In the absence of p53, katanin expression is strongly reduced and microtubule organization is disturbed, suggesting a linkage between hypoxia and microtubule stability. Katanin p60 was shown to regulate the spindle pole during cytokinesis and its inhibition caused an incomplete cell division (Matsuo et al., 2013). Nevertheless, it is controversial that clinical observations indicated a function of katanin as a tumor promoter, but the regulation of p53 on katanin transcription provided an opposite activity. It has been reported that katanin overexpression inhibited cell growth but promoted metastasis (Fu et al., 2018). It possibly depended on the dominant regulators facilitating transcription or post-translation of katanin under specific circumstances and the cellular responsiveness to the tumor microenvironment.

## Conclusion and perspectives

Cancer is a life-threatening disease, mainly caused by its ability to metastasize. The eradication of metastatic cancer cells remains a challenge in anti-cancer drug research and discovery. Due to its complex process, incorporating various signaling pathways, which are largely unidentified, several attempts have been made to identify key players as potential therapeutic targets. Microtubules are fundamental cytoskeleton components, participating in various cellular activities, including supporting cell morphology, chromosome segregation, cell motility, and trafficking. Although microtubules are found in both normal and malignant cells, some differences have been identified in microtubule behavior, dynamics, and post-translational modifications (Wattanathamsan and Pongrakhananon, 2021), which MAPs mediate. The biological role of MAPs has gained increasing interest. Importantly, several MAPs have expression and/or mutation profiles distinctive from normal tissue, rendering them value as potential predictive markers.

Based on the classical function of microtubules, some MAPs regulate cancer metastasis through microtubule reorganization and/or microtubule dynamic alterations. For example, microtubule disruption by microtubule-severing proteins can disturb cell migratory activity. The enhancement of microtubule stability or post-translational modifications can alter the trafficking of signals related to metastasis. Besides, emerging evidence has shed light on the direct mechanism whereby MAPs are key drivers regulating metastasis. Many MAPs have direct effects on signaling pathways independently from their microtubule association, likely by altering the expression of signaling proteins. At present, little is known about the molecular mechanism of MAPs, and their further identification might provide us with predictive/prognostic markers and/or therapeutic targets, thereby improving clinical outcomes.

Recently, MAPs have been discovered as biomarkers for drug sensitivity of metastasis tumors, particularly taxane-based chemotherapy. Transcriptomic analysis from breast cancer patients treated with taxanes showed that some of the genes encoding MAPs were differently expressed, and one of the interesting genes was MTSU1, which encodes ATIP3. The expression of ATIP3 was decreased in taxane-sensitive breast tumors (Rodrigues-Ferreira et al., 2019), and downregulation of MTSU1 was associated with low lymph node metastasis after obtaining taxane-based chemotherapy (Rodrigues-Ferreira et al., 2020). MAPT was also downregulated in ovarian cancer patients who had well responsive to paclitaxel (Smoter et al., 2013). Besides, breast cancer patients with a lower level of STMN1 displayed a higher sensitivity to docetaxel-containing chemotherapy (Meng et al., 2012). These studies provide intriguing biomarkers for predictive chemotherapy responsiveness, and substantial experiments for validation may contribute to a therapeutic approach for restoring the resistance to taxane-based chemotherapy, facilitating the translation of research to clinical application.

Currently, drugs targeting MAPs are under the drug design process, while some are in clinical trials. One upcoming strategy is to interact with and suppress MAP’s function that regulates microtubules participating in cancer cell division. The quinazolinone-based kinesin inhibitors were in clinical phases 1-2. Mode of action is involved with the inhibition of kinesin binding to microtubules, thereby suppressing mitotic spindle assembly and mediating cell cycle arrest (Matsuno et al., 2008). Ispinesib mesylate selectively inhibits kinesin spindle protein (KSP) which is under phase II clinical trial for breast cancer treatment (Khwaja et al., 2021). The other KSP inhibitors, such as MK-0731, SB743921, and ARRY-520, are under clinical trial in solid tumors (Holen et al., 2012; Khoury et al., 2012; Khwaja et al., 2021). The second strategy is to target MAPs associated with microtubule stability. Since taxane- and vinca alkaloid-based drugs that bind to and interfere with microtubule stabilization have low clinical outcomes due to drug resistance after prolonged regimens, MAPs regulating microtubule stability become attractive targets in drug discovery. GRC0321, a purine-based compound, was sought to be a katanin-targeting agent, which showed a potent apoptosis-inducing effect in lung cancer cells (Kuo et al., 2016). These purine-type agents directly target katanin via stabilizing the katanin p60 hexamer and inducing its microtubule-severing activity. Recently, compound 20b was identified targeting katanin. Compound 20b prevented cancer cell proliferation by interacting with the katanin in non-small cell lung cancer (Gao et al., 2019). As an aberrant expression in cancers, the target at katanin may provide an advantage in the aspect of selectivity to cancer with a lower effect on normal cells. However, this concept requires verification.

Another drug design and development strategy is hybrid drugs that have multiple modes of action such as disrupting microtubule dynamics in combination with another anti-cancer mechanism. This strategy is a rationally attractive approach to enhance efficacy and reduce severe toxicity, in which hybrid drugs are a combination of at least two pharmacophores with covalent linkage in a single molecule to cooperate with multiple targets. Hybridized microtubule targeting drugs were developed including combretastatin hybrids, vinca alkaloid hybrids, and a-noscapine hybrids (Gherbovet et al., 2014; Vilanova et al., 2014; Mahaddalkar et al., 2016; Devi Tangutur et al., 2017). Although this strategy first came up for interrupting microtubule dynamics, this concept provides a more expandable approach for drug design targeting MAPs. Recently, arylaldoxime/5-nitroimidazole hybrids were synthesized to inhibit microtubule affinity-regulatory kinase 4 (MARK4), along with exerting anti-oxidant activity (Peerzada et al., 2020). MARK4 belongs to the serine/threonine kinases family that is required for phosphorylating MAPs, including MAPT and MAP2, thereby affecting the microtubule dynamics (Trinczek et al., 2004). As MARK4 promotes cancer progression (Heidary Arash et al., 2017) and oxidative stress associated with cancer pathogenesis (Zahra et al., 2021), the molecular hybridization of this molecule provided a significant anti-cancer activity. However, the anti-metastatic activity of these agents requires further investigation. Hence, we can at least infer that MAPs are promising therapeutic targets for anti-cancer drug discovery and development.

Microtubule post-translational modifications (PTMs) are implicated as a mechanism underlying the MAP-mediated regulation of cancer behaviors. Generally, microtubule PTMs are maintained in a balanced state and play a role in cellular activity, in which they are regulated by several enzymes. One such enzyme is histone deacetylase 6 (HDAC6), which is localized predominantly in the cytoplasm and controls nonhistone acetylation, such as that of α-tubulin (Li C. et al., 2018). High expression levels of HDAC6 have been detected in numerous types of cancer and such expression is strongly associated with a poor overall survival rate (Zhang et al., 2017; Li T. et al., 2018). HDAC6 regulates essential biological processes in cancer through α-tubulin deacetylation (Li C. et al., 2018). Additionally, HDAC6 was reported to participate with MAPs, including CLIP-170 and CAMSAP2, in promoting cancer metastasis (Li et al., 2014; Li et al., 2020). HDAC6 inhibition is, therefore, one possible approach in cancer therapy. Several selective HDAC6 inhibitors are now the subject of clinical trials for cancer treatment (Pulya et al., 2021). Ricolinostat (ACY-1215) is well-recognized as monotherapy or can be used in combination with dexamethasone, bortezomib, or lenalidomide in refractory or relapsed multiple myeloma (Vogl et al., 2017). Citarinostat (ACY-241), the second-generation HDAC6 inhibitor, is an orally active HDAC6 inhibitor with improved potency (Niesvizky et al., 2015). Given that its pharmacophores have been identified, the selectivity and efficacy of HDAC6 inhibitors are of interest concerning further drug design and development.

Although the roles of MAPs have been explored in some respects, our scientific understanding of MAPs can still be improved and validated. Nevertheless, it is now clear that some MAPs, such as MAPT, ATIP3, and STMN1, as well as enzymes that regulate microtubule behaviors, such as HDAC6 and MARK4, are potentially important cancer therapeutic targets and/or predictive/prognostic biomarkers. Furthermore, the development of molecular hybrids as microtubule- or MAP-targeting agents is in progress, and such agents will improve drug efficacy and selectivity while reducing side effects. Overall, this review collects the recent important evidence that will help guide researchers conducting further research on MAPs in cancer.
